# Effective Preventive Strategies to Prevent Secondary Transmission of COVID-19 in Hemodialysis Unit: The First Month of Community Outbreak in Taiwan

**DOI:** 10.3390/healthcare9091173

**Published:** 2021-09-07

**Authors:** Chun-Yu Chen, Jung-Jr Ye, Ting-Shuo Huang, Chin-Chan Lee, Yih-Ting Chen, Cheng-Kai Hsu, Heng-Jung Hsu, Chiao-Yin Sun, Heng-Chih Pan, Kuo-Su Chen, Hao-Hsi Kao, Chia-Chun Ko, Yun-Hsuan She, Chun-Ying Wu, Chi-Chun Lai, Shang-Jyh Hwang, I-Wen Wu

**Affiliations:** 1 Department of Nephrology, Chang Gung Memorial Hospital, Keelung 204, Taiwan; shone@cgmh.org.tw (C.-Y.C.); leefang@cgmh.org.tw (C.-C.L.); b9402031@cgmh.org.tw (Y.-T.C.); kylegb@cgmh.org.tw (C.-K.H.); r5267@cgmh.org.tw (H.-J.H.); sun3970@cgmh.org.tw (C.-Y.S.); balour@cgmh.org.tw (H.-C.P.); cksdavid@cgmh.org.tw (K.-S.C.); luhuichun@cgmh.org.tw (H.-H.K.); kinanna0815@cgmh.org.tw (C.-C.K.); she.yun.hsuan@gmail.com (Y.-H.S.); 2 College of Medicine, Chang Gung University, Taoyuan 333, Taiwan; ccl404@cgmh.org.tw; 3 Department of Infectious Diseases, Chang Gung Memorial Hospital, Keelung 204, Taiwan; loyalwise@cgmh.org.tw; 4 Community Medicine Research Center, Department of General Surgery, Chang Gung Memorial Hospital, Keelung 204, Taiwan; huangts@cgmh.org.tw; 5 Department of Chinese Medicine, College of Medicine, Chang Gung University, Taoyuan 333, Taiwan; 6 Department of Laboratory Medicine, Chang Gung Memorial Hospital, Keelung 204, Taiwan; hla0861@cgmh.org.tw; 7 Department of Ophthalmology, Chang Gung Memorial Hospital, Keelung 204, Taiwan; 8 Department of Internal Medicine, Division of Nephrology, Kaohsiung Medical University Hospital, Kaohsiung 807, Taiwan; sjhwang@kmu.edu.tw; 9 Faculty of Renal Care, College of Medicine, Kaohsiung Medical University, Kaohsiung 807, Taiwan

**Keywords:** COVID-19, incidence, mortality, outcome, prevention, strategies

## Abstract

Background: Dialyzed patients are vulnerable to coronavirus infection disease 2019 (COVID-19). The incidence and outcome of COVID-19 in hemodialysis (HD) patients in Taiwan remain unclear. A series of preventive measures were executed to combat COVID-19 transmission among HD patients. Methods: We carried out a series of forward-looking and practical preventive strategies of COVID-19 control in our HD center. Incidences of COVID-19 of our HD unit were compared with those of national and local estimates from a community outbreak from 15 May to 30 June 2021. Prognostic factors associated with mortality were analyzed. Results: The national incidence of COVID-19 was 0.062%; being highest in Taipei City (0.173%), followed by New Taipei City (0.161%) and Keelung (0.083%). The overall incidence in Keelung HD patients was 0.666%. One patient of our HD center contracted COVID-19 from the household; however, we have contained secondary transmission in our HD center by implementing strict preventive measures. The mortality rate of HD patients in Keelung was 66.6%. The median Ct value of HD patients was 17.53 (11.75–27.90) upon diagnosis. The deceased patients had a higher cardiac/thoracic ratio than alive (0.61 vs. 0.55, *p* = 0.036). Conclusions: Taking aggressive and proactive infection preventive measures impedes the secondary transmission of COVID-19 in HD facilities. COVID-19-associated mortality was high in HD patients, being the high cardiac-thoracic ratio, an important prognostic factor for clinical outcome of infected HD patients.

## 1. Introduction

The 2019 novel severe acute respiratory syndrome coronavirus 2 (SARS-CoV-2) infection related disease (COVID-19) is extremely contagious, sometimes lethal in high-risk patients, and was declared a global emergency affecting 2.53% of the 7.8 billion population worldwide [1]. The disease is continuing to engender a tremendous global healthcare burden up to today. COVID-19 infection, which originated in Wuhan City of China in December 2019, has quickly spread to all continents, causing 197,825,365 confirmed cases and 4,216,780 deaths worldwide to 1 August 2021 [1]. Fortunately, the implementation of universal prevention and vaccination policies has led to a substantial reduction in the number of newly diagnosed cases in some countries, such as the United States, Israel, and the United Kingdom. However, the mortality rate remains high in other nations, including the United States, Brazil, and India [1]. Taiwan has fought against the disease and has achieved successful containment of the infection keeping up to 253 days of zero confirmed domestic cases since the beginning of the overwhelming global situation of the pandemic in 2020 [2]. The dissemination of infection has been prevented by using vigorous national control measures, such as country border restrictions, proactive case identification using electronic contact tracing, quarantine of suspicious and confirmed cases, enhancing hygiene and sanitation, mandatory masking, social distancing, healthcare resource allocation, and efforts in reassurance and education of citizens [3,4,5]. However, a second-wave outbreak of the COVID-19 pandemic has emerged in North Taiwan since 14 May 2021 and has spread into other regions of the country. Taiwan is attacked majorly by SARS-CoV-2 variant B.1.1.7 which originated from the United Kingdom; however, a small proportion of the delta variant, also known as B.1.617.2, was also noted [6]. The two virus variants are more transmissible, more virulent, and may trigger more grave clinical conditions than non-variants lineage [7,8]. As of 30 June, Taiwan has reached 14,804 laboratory-confirmed SARS-CoV-2 infection cases with an increasing death toll to 648 fatalities, especially in two hardest-hit areas, Taipei City and New Taipei City [9]. However, this community outbreak has been rapidly controlled nationwide. Sharing these experiences can certainly help in designing effective COVID-19 control measures in areas of similar socio-economic or epidemiological conditions.

End-stage renal disease (ESRD) patients represent a vulnerable population with increased risks for COVID-19 infection and for related death [10]. In-center hemodialysis (HD) is currently the most common renal replacement therapy modality for ESRD patients worldwide [11]. The regular needs of visiting a medical facility to receive their dialysis treatment, often confined in crowded spaces, make it difficult to comply with some of the effective protective measures, such as stay-home orders, social distancing, and reducing personal contacts. The risks of mortality in SARS-CoV-2 infected people include older age, presence of comorbidities, and immune dysfunction [12,13]. HD patients are often elderly and immune-dysregulated, owing to uremia, underlying comorbidities, and dialysis procedure-related biocompatibility and micro-inflammation [14]. Therefore, specific preventive methods to protect HD patients from COVID-19 infection remain urgently needed.

Experiences of effective preventive strategies to combat secondary transmission of COVID-19 in HD units remain limited. Keelung City, 30 km away from the hotspot area of the pandemic, has become a region of alert for the prevention of community spreading of the disease. Taking appropriate measures immediately in the HD unit is the top priority to hamper the dissemination of the disease. The common preventive strategies adopted in the HD unit comprise the use of independent ward or cohort isolation, hand sanitizing, quarantine, and effective personal protective equipment [10]. However, the infection rate remains still high in HD units in spite of the application of all these methods [10]. Taiwan has the highest prevalence of ESRD worldwide [11]; however, the estimation of incidence and outcome of COVID-19 infection in Taiwanese HD patients is unclear. In spite of national and official public health policies to fight against COVID-19, effective preventive strategies to impede the secondary transmission of COVID-19 in HD units remain to be established. The aim of this study was to design a series of preventive measures to fight against the spreading of SARS-CoV-2 infection among HD units and to evaluate the effectiveness of these measures on the incidence of COVID-19 and related prognosis of HD patients in this era of crisis. The information could be mandatory to limit the infection and dissemination of COVID-19 in HD facilities.

## 2. Materials and Methods

### 2.1. Study Design and Subject Characteristics

This study was conducted in a single hospital-affiliated HD center of Chang Gung Memorial Hospital at Keelung City, the largest HD unit of north-east Taiwan having 120 beds and caring for 497 maintenance HD patients. All of these patients were followed from the beginning of the community outbreak on 14 May 2021 to 30 June 2021. Demographics, laboratory data, and the incidence of COVID-19 were accurately recorded. All patients must comply with national public health preventive measures for the COVID-19 pandemic from the Taiwan Center for Disease Control (CDC) and several additional specific preventive strategies promulgated by our HD center. They have to agree to report their traveling fingerprint, including their traveling, occupational, contact, and clustering history (TOCC). The enrolled patients should be vaccinated in compliance with the government’s COVID-19 vaccination program, except those with contraindications to vaccines. This study was performed in accordance with the Declaration of Helsinki and was approved by the Ethics Committee of the Institutional Review Board at Chang Gung Medical Foundation (IRB: 202100854B0A3).

### 2.2. Formation of COVID-19 Joint Rapid Response Team

A COVID-19 joint rapid response team from the HD Center in Chang Gung Memorial Hospital at Keelung was formed immediately after the outbreak of COVID-19 in Northern Taiwan on 15 May. The team included experts of nephrology and infectiology, leaders of HD nursing staff, nephrology nursing practitioners, members of the committee of infection control, and the chief person of the Administrative Department. All members of this multidisciplinary team joined the Team Pro^®^ and LINE^®^ group to facilitate immediate communication. The directors of the nephrology and infectiology department led the team and headed this collaborative effort. The COVID-19 joint rapid response team formulated our exclusive COVID-19 clinical infection preventive guidelines for HD patients and staff, arranged the acceptance of confirmed SARS-CoV-2 infection HD patients from outside clinical HD facilities, and connected extensively with the Department of Health of Keelung City Government to update daily infected cases. On 17 May 2021, the joint response team announced the first draft of the COVID-19 clinical practice guidelines for our HD Center, which were on the basis of recommendations from the Taiwan CDC and Taiwan Society of Nephrology (TSN) [15,16,17,18]. Our guidelines were targeted to prevent COVID-19 infection of HD patients, to minimize secondary spreading, and to establish response methods of our HD Center against the disease (Table 1).

### 2.3. Specific Preventive Strategies in HD Center of Chang Gung Memorial Hospital at Keelung

**Figure 1:**
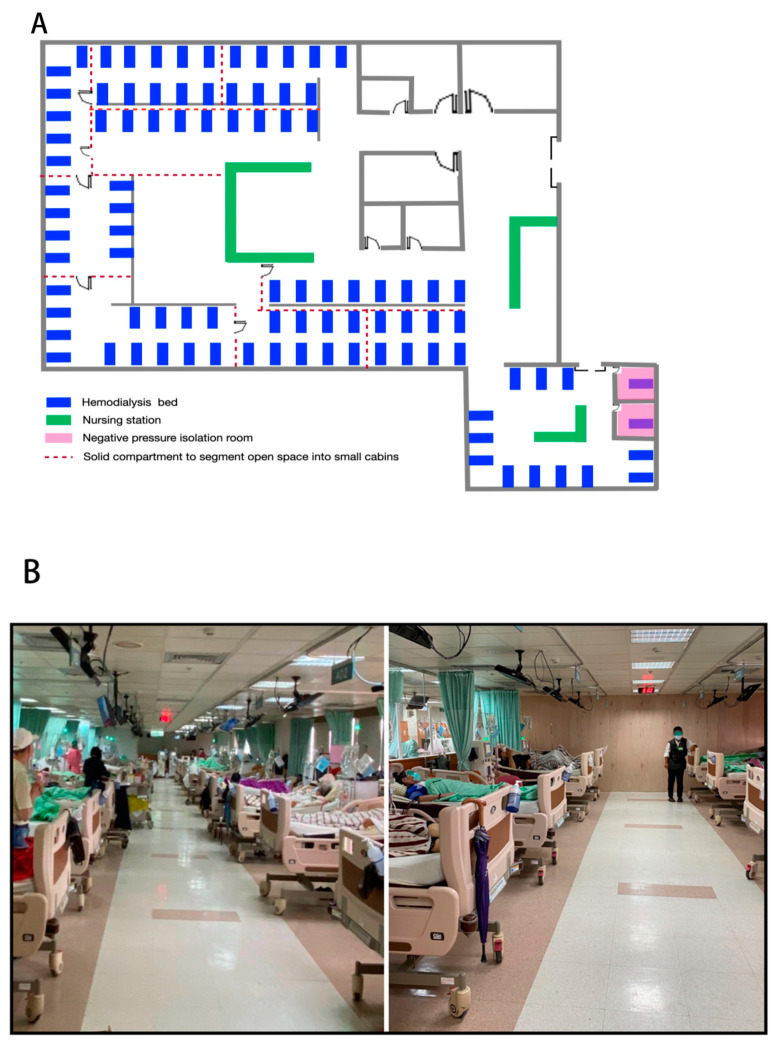
Space segmentation of HD unit of Chang Gung Memorial Hospital at Keelung. (**A**) Aerial view of space segmentation; (**B**) use of solid wood compartment to separate the open room into small cabins (left, before; right, after segmentation).

**Figure 2:**
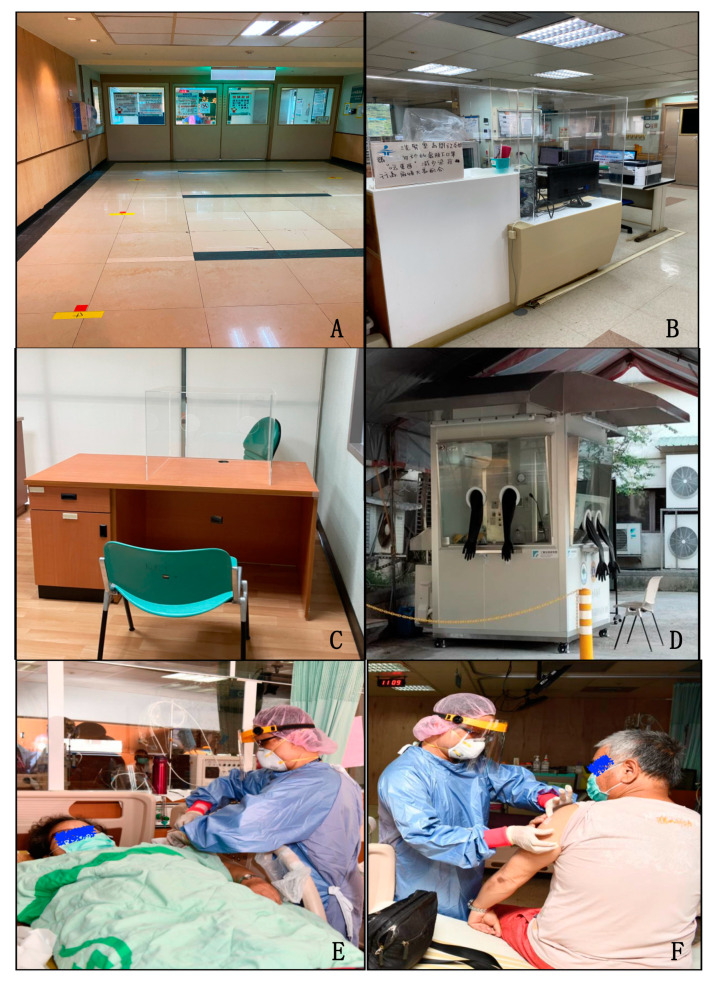
Specific preventive measures for COVID-19 infection control in the HD unit of Chang Gung Memorial Hospital at Keelung. (**A**) Set up position mark at entry of HD unit; (**B**) acrylics compartment for nursing station to prevent droplet dissemination; (**C**) weekly rapid test for early detection of asymptomatic patients at temporary setting or (**D**) permanent settings; bedside vaccination at HD unit during supine (**E**) or setting position (**F**).

**Figure 3:**
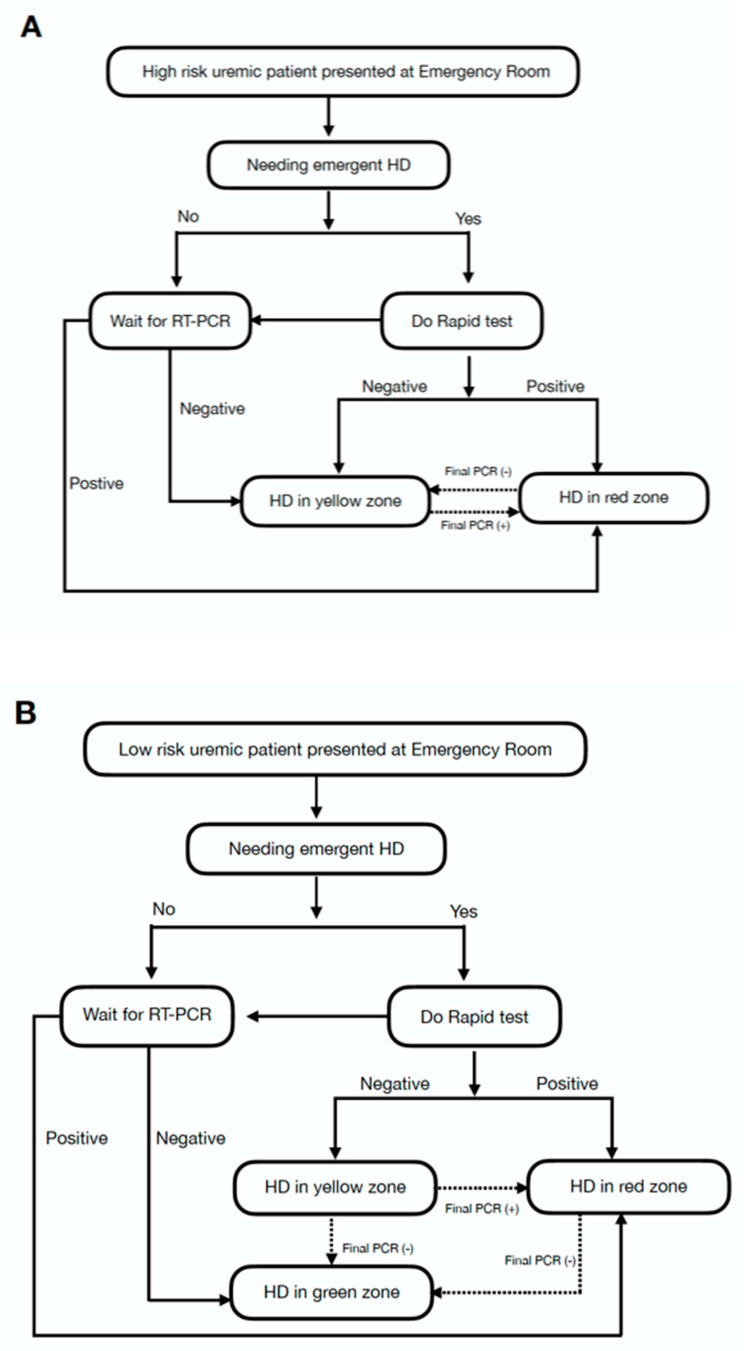
Triage algorithm of emergent patients needing HD during pandemics in Chang Gung Memorial Hospital at Keelung. (**A**) Algorithm for patients with high-risk of COVID-19, defined as having (1) recent home quarantine or self-health management (for less than 21 days), (2) community pneumonia, (3) resident of institutions, (4) any fever (ear temperature ≥ 38 degrees), respiratory symptoms or diarrhea or abnormalities in taste or smell sensation, (5) recent exposure to confirmed patients; (**B**) algorithm for patients with low-risk of COVID-19 (who do not meet any of the abovementioned criteria).

The preventive guidance issued by the TSN instructed universal measures for patients and medical staff to combat COVID-19, complimenting those interventions of the CDC [15,16,17,18]. The measures for patients were comprised of portal control, TOCC tracking and triaging, mandatory masking, health status, and temperature monitoring, restriction of eating and drinking in the treatment area during the whole HD session and limiting caregivers. In addition to all these measures, effective personnel protection equipment (PPE, using goggles, masks, gloves, isolation gowns) and frequent alcohol hand disinfection were recommended for all medical and nursing staff. For workplace safety, use of independent treatment rooms, appropriate environmental and equipment disinfection, especially in frequently touched areas by multiple people (toilet, doorknobs, elevator buttons, and others), maintenance of air-conditioning, and good ventilation conditions were suggested [16,19]. In spite of the use of these measures, outbreaks of COVID-19 infection were noted in different HD facilities nationwide. Furthermore, we implemented additional strategies to prevent the spreading of infection in our HD Center, focusing on the following aspects: space segmentation, access control, quarantine operations, and medical staff protection (Table 1). Most of the HD units used curtain separation rather than solid compartments between patients during their dialysis sessions. In addition to this universal measure, we utilized wood, strengthened glass, and acrylic materials to building small cabins (of 8–10 beds). This construction was safe, affordable, and rapid to reduce potential contacts and exposure to infected cases from the open space of the HD room (Figure 1). The entry to the HD unit, at each beginning of the treatment session, can cause clustering of patients in a crowded area during a concentrated period of time, which increases the risk of personal contacts and represents an important shortcoming for infection prevention. To overcome this disadvantage, we have established pacing between exchanges of HD sessions to avoid overlapping of patients at the entry and exit of HD rooms and have installed position marks on the ground of the entrance, with a 1.5 m distance between patients to avoid congregation (Figure 2A). We also installed acrylic partitions in the nursing station to limit droplet and aerosol dispersion (Figure 2B). Weekly rapid nasopharyngeal swab SARS-CoV-2 antigen testing was mandatory for all HD patients and fixed caregivers to promote early detection of asymptomatic cases. This maneuver has been conducted in an independent negative pressure room with the use of an auxiliary acrylic box and performed by physicians wearing complete PPE (Figure 2C) until a permanent positive-pressure cabinet became available (Figure 2D). The real-time PCR (RT-PCR) nucleic acid test was performed if a positive result from the rapid antigen test was noted. Suspected cases (or those waiting for RT-PCR results) received HD sessions in a negative pressure room via a separate gateway to enter our HD unit wore an N95 mask during the treatment session. All suspected cases were instructed to avoid public transportation until receiving negative RT-PCR results. For confirmed cases, patients were hospitalized in an independent negative pressure room and received bedside HD via portable reverse osmosis (RO) system. The patients received at least 14-days of hospitalization until a negative RT-PCR result became available. Vaccination was highly promoted to all HD patients. We offered bedside inoculation at the end of their HD treatment. The beside vaccination program can incentivize the willingness to receive a vaccine and can also reduce further clustering at the vaccination station (Figure 2E,F). To avoid possible contamination of asymptomatic cases to our HD unit, we also developed triage algorithms, according to COVID-19 risk stratification, to manage those uremic patients presenting in the emergency room (ER) needing HD therapy (Figure 3). COVID-19 clinical practice guidelines raised by our joint rapid response team further instructed patients and staff to comply with all these measures. Periodic surveillances in the adherence of these interventions were conducted by a third party of the administrative department.

### 2.4. Adjustment of Preventive Measures According to Reproduction Number (R_t_)

The transmission rate of COVID-19 infection in specific settings relies on the overall prevalence of disease of the region and can be estimated by the effective reproduction number (Rt) [20]. It is defined as the mean number of secondary cases generated by a typical primary case at a given time in a population and can be calculated for the whole period over a 5-day moving average [21]. The effective Rt can be used to evaluate the effectiveness of public health measures and should be quantified in different settings at regular and frequent intervals [22,23]. We adjusted our preventive plans according to the overall prevalence of COVID-19 in Keelung. We also stepped down our preventive measures guided by the Rt values (national estimates and those of the three high prevalent pandemic areas: Taipei City, New Taipei City, and Keelung) to achieve smooth recovery of normal medical functioning.

### 2.5. Laboratory Confirmation for SARS-CoV-2 Infection: Rapid Antigen Test and RT-PCR Analysis

We used the VTRUST COVID-19 Antigen Rapid Test (TaiDoc company, Taiwan) to detect SARS-CoV-2 nucleocapsid protein by using lateral flow chromatographic immunoassay on a weekly basis. The samples were obtained by nasopharyngeal swab specimen and results were obtained in 15 min. The limit of detection (LoD) was 1.26 × 102 TCID50 per mL and the sensitivity and specificity were 93.1% and 99.6%, respectively. Rapid test positive cases were further ascertained by nucleic acid testing using RT-PCR. Specimens for RT-PCR were obtained through nasopharyngeal swabs and immersed into 0.3 mL of preservation solution containing 30 μL proteinase K and 0.33 mL VXL buffer. The multiplex RT-PCR (LabTurbo™ AIO COVID-19 RNA testing kit) was applied to detect SARS-CoV-2 RNA by using a full-process automated system. The multiplex reaction detected COVID-19 N1 gene, coronavirus E gene, and RNase P gene (internal control) in one single well. The COVID-19 RdRP gene was used for reconfirmation of the detection. The primer and probe sequences for RNA-dependent RNA polymerase gene detection were described as GTGARATGGTCATGTGTGGCGG (forward), CAAATGTTAAAAACACTATTAGCATA (reverse), and FAM-CAGGTGGAACCTCATCAGGAGATGC-BBQ (probe). Meanwhile, the primer and probe sequences used for E gene detection were ACAGGTACGTTAATAGTTAATAGCGT (forward), ATATTGCAGCAGTACGCACACA (reverse), FAM-ACACTAGCCATCCTTACTGCGCTTCG-BBQ (probe). The primer and probe sequences used for the N1 gene detection were as follows: GACCCCAAAATCAGCGAAAT (forward), TCTGGTTACTGCCAGTTGAATCTG (reverse), FAM-ACCCCGCATTACGTTTGGTGGACC-BBQ (probe). The cycle threshold (Ct) values of RT-PCR were converted into RNA copy number of SARS-CoV-2. The RNA copy number was calculated from a standard curve on the basis of the Ct values of plasmid DNA. The LoD of the quantitative PCR reaction for the N1 gene was 1 copy/μL, for the RdRP gene was 1 copy/μL, for the E gene was 2 copies/μL, and for the RNaseP gene was 0.1 copy/μL. Specimens were considered as negative if the Ct values exceeded 36 cycles. The technique for COVID-19 RNA detection is approved by the Taiwan Food and Drug Administration with emergent use authorization (EUA, approval number: 1096817658).

### 2.6. Data Source of Local and National Burden

The number of nationally confirmed cases and those of high prevalent areas, including Taipei City, New Taipei City, and Keelung, were recorded. The number of laboratory-confirmed SARS-CoV-2 infections in Taiwan and of the individual counties or cities were obtained from the Taiwan CDC [9]. Information of COVID-19-infected HD patients in Keelung was obtained from the Taiwan CDC and the Daily Press conference of the Keelung City Government. The numbers of actual confirmed cases and the 7-day moving average confirmed cases were recorded for the analyses.

### 2.7. Statistical Analyses

Descriptive statistics were expressed as the mean (standard deviation) or median (interquartile range). Discrete variables were presented in terms of frequencies and percentages. The normality of numerical variables was tested using the Kolmogorov–Smirnov method, and an appropriate transformation was considered before statistical testing. The Student’s *t*-test or Mann–Whitney U-test was applied to compare the mean or median values among the groups. The association between categorical variables was analyzed using the chi-square test. We estimated the Rt values using the methods provided in the R package (EpiNow2, ver. 1.3.3) [21]. All statistical tests were two-tailed, and a *p*-value < 0.05 was considered to be statistically significant. Data were analyzed using the statistical package from the social sciences software (SPSS, Inc., Chicago, IL, USA) version 26.0 for Mac.

## 3. Results

### 3.1. Study Subject Characteristics

Table 1 summarizes the clinical characteristics of our HD patients. The mean age was 66.03 ± 13.28 years, comparable to the average age of the national HD patients starting HD. The mean hemoglobin was 10.06 ± 1.25 g/dL and the Kt/V was 1.67 ± 0.33. The overall nutrition status was acceptable with a mean normalized protein catabolic rate of 1.08 ± 0.39 g/kg/day and albumin of 4.00 ± 0.48 g/dL, respectively. The only SARS-CoV-2-infected HD patient was younger (60.8 years), had less hemoglobin (8.20 g/dL), less platelet count (173 × 1000/uL), less albumin (3.76 g/dL), less cholesterol (139 mg/dl), and higher intact-parathyroid hormone (440 pg/mL) values (Table S1).

### 3.2. An Overview of the Pandemic in Northern Taiwan in the First Month of Outbreak

**Figure 4:**
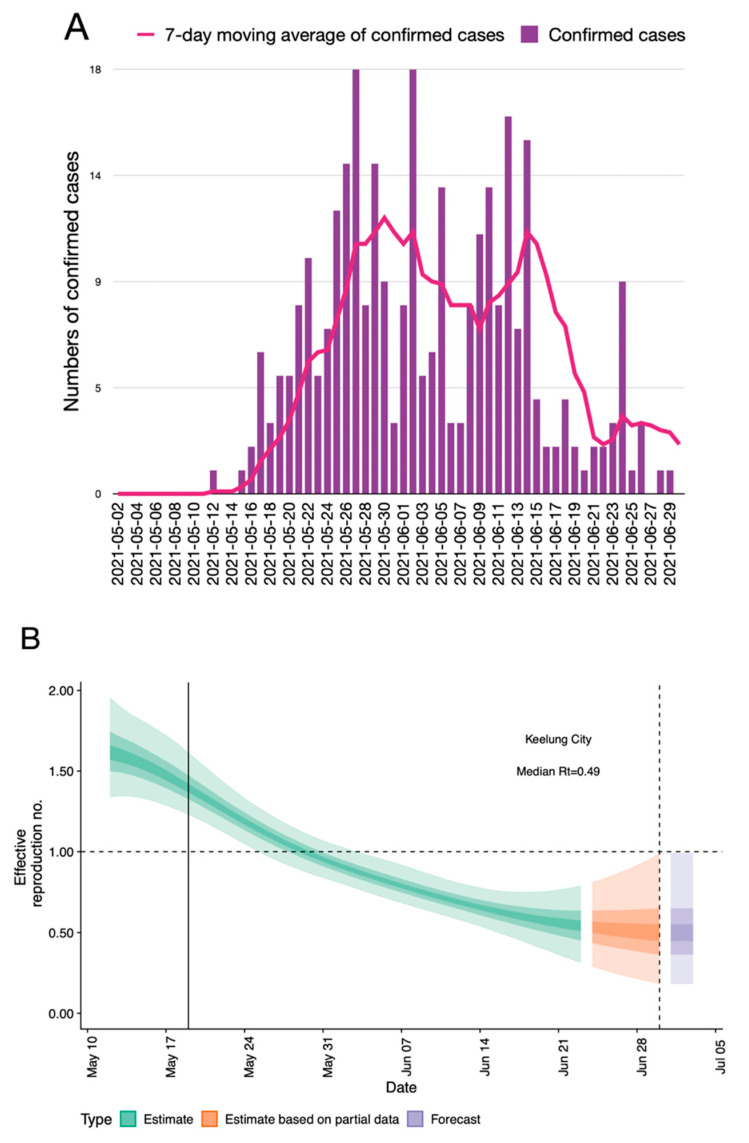
Estimates of COVID-19 confirmed case numbers and 7-day moving average of confirmed cases in Keelung (**A**). Estimated and predicted time-varying reproduction number (Rt) of SARS-CoV-2 with 20%, 50%, 90% credible intervals in Keelung (**B**).

Taiwan has maintained zero domestic infections for 253 days and had 12 deceased patients in 2020. However, a cluster of COVID-19 cases occurred in a hotel in Taoyuan County at the end of April 2021, imported by aircraft crews. After that, COVID-19 rapidly spread to northern Taiwan and finally broke out on 14 May 2021, with daily 185 newly diagnosed domestic cases. Taiwan has accumulated 14,804 confirmed cases and 648 deaths as of the end of June (Figure S1A). The northern cities were hit hardest by the pandemic. The national incidence of COVID-19 was 0.062%; being highest in Taipei City (0.173%), followed by New Taipei City (0.161%) and Keelung (0.083%), as of 30 June (Table 2). The overall incidence of COVID-19 in Keelung HD patients was 0.666%. One single HD patient contracted the COVID-19 infection from the household; however, we were able to contain secondary transmission of this case to our HD unit by using strict implementation and adherence to preventive measures. This community outbreak peaked in the period of 22–30 May in Taipei City, having daily 550 new confirmed cases (Figure S1B), and subsequently spread out to New Taipei City (Figure S1C) and Keelung. Bimodal waves of the pandemic were observed in Keelung with clustering in several marketplaces (Figure 4A). Fortunately, the pandemic has been contained with decremental cases nationwide over time (Figure S2A).

### 3.3. Outcomes of Strict Implementation of Specific Preventive Strategies in HD Unit

Prevention of nosocomial spreading of airborne diseases, such as COVID-19, remains difficult in HD settings, especially in hotspot areas. To overcome this barrier, we have established specific preventive strategies for patient and staff safety (Table 3). One single patient of our HD unit contracted the COVID-19 infection from her family members. Confirmed HD cases or those with exposure to confirmed cases were moved to negative pressure isolation rooms for their dialysis treatment. Close contacts of these cases, including nursing staff and surrounding HD patients were all tested for RT-PCR. In spite of negative RT-PCR results, the nursing staff received home quarantine for two weeks and the surrounding HD patients were placed in a separated area for dialysis for 14 days. Fortunately, no new confirmed cases were noted after that episode, and we have successfully contained any secondary transmission of COVID-19 among our HD patients by using advanced specific preventive measures (Table 3). Although all of these measures were efficient and were rapidly implemented at the beginning of the outbreak; however, the interventions were both manpower- and resource-consuming and may hamper many other functioning of normal medical practice. The uses of our preventive measures were adjusted according to the regional prevalence and Rt values of the endemic region. The Keelung area exhibited the greatest reduction of Rt values over time (Figure 4B). Consequently, several measures (such as weekly rapid testing of our HD patients and caregivers) were suspended at six weeks after the initiation of the outbreak when the overall prevalence of Keelung dropped and the vaccination rate of our patients had reached more than 80% of the whole HD patient pool.

### 3.4. Outcomes of COVID-19 Confirmed HD Patients

Two of the 497 HD patients had persistent positive weekly rapid test results for more than 4 weeks; however, no presence of fever or clinical symptoms were noted in these two cases. The weekly RT-PCR testing for them was all negative. One single confirmed patient of our HD unit transmitted from the household developed severe pneumonia, while she responded well to therapy, survived, and was eventually discharged. In contrast, eight confirmed HD cases of infection were noted in a neighborhood HD unit at Keelung using only universal preventive measures of the CDC (Table 2). One of these patients died prior to admission to our hospital and five of them died after admission to our intensive care unit. Table 4 shows the characteristics of SARS-CoV-2 infection confirmed HD patients of Keelung City admitted to our hospital. Overall, nine confirmed HD cases were found at Keelung and the mortality rate was 66.6% (6 of 9). The median C_t_ value of hospitalized patients was 17.53 (11.75–27.90) upon diagnosis. The deceased patients tend to be older (74.9 vs. 63.1 years, *p* = 0.143), more likely having a higher cardiac/thoracic ratio (0.61 vs. 0.55, *p* = 0.036) and higher serum C-reactive protein (52.8 vs. 17.2 mg/L, *p* = 0.0711). The serum levels of lactate (97.5 vs. 11.05, *p* = 0.333), procalcitonin (0.56 vs. 0.53 ng/mL, *p* = 0.393), and C_t_ value (17.47 vs. 17.53, *p* = 1.000) did not differ between deceased and alive patients, respectively (Table 4). All patients died from respiratory failure due to pneumonia having a median survival time of 13 (8–19) days from the date of diagnosis.

## 4. Discussion

In the present study, we have demonstrated that taking aggressive and proactive infection preventive measures could successfully impede secondary transmission of COVID-19 in HD facilities. The nosocomial spreading was greater in HD units than in other settings because of the high susceptibility of HD patients to SARS-CoV-2 infection. This vulnerability was inherent to the particular clinical- and treatment-related characteristics of dialysis patients. The national government has issued a level-3 alert since 17 May and has suspended school classes, limited the number of gatherings, prohibited businesses in entertainment venues, and forbidden meals in restaurants. Our COVID-19 joint rapid response team managed COVID-19 control through collaborative activities and has formulated clinical infection preventive guidelines for our HD center from the very early stage of the outbreak. In addition to national preventive measures, strict implementation and adherence of four major specific strategies: space segmentation, access control, quarantine operations, and high-quality medical staff protection have shown advantages to fighting against SARS-CoV-2 infection and have ensured the safety of patients and staff during their hemodialysis treatment, even in the presence of confirmed cases. However, COVID-19-associated mortality is high in HD patients, being the high cardiac-thoracic ratio, an important prognostic factor for clinical outcomes of infected HD patients.

Coronaviruses, commonly transmitted human-to-human, have caused several disastrous pandemics in human history. The outbreak of severe acute respiratory syndrome (SARS) in 2003 resulted in 346 confirmed SARS cases and 37 deaths in Taiwan. Nonspecific clinical presentation, absence of accurate and timely diagnostic methods, as well as a lack of experience of national prevention control measures in facing an emergency of a highly contagious airborne disease has led to nosocomial transmission and a catastrophic outbreak of the SARS epidemic in Taiwan [24]. Both SARS and Middle East respiratory syndrome (MERS) coronavirus infections have never contributed to secondary transmission in HD facilities [25,26,27]. Similarly, the prevalence of COVID-19 among HD settings has been higher than in the general population. Our previous systematic review and meta-analysis study has revealed that the overall incidence of COVID-19 in HD patients was 7.7%, the incidence rate being lower in Asian HD patients (5.0%, 95% CI: 2.5–8.4%) than those of non-Asian countries (10.5%, 95% CI: 6.6–15.3%) [10]. Although the national estimates of the incidence of COVID-19 in HD patients remain unclear, we have shown that the overall incidence rate of COVID-19 confirmed HD patients at Keelung was 0.666%, 10-fold higher than the national COVID-19 infection estimate of the general population. Episodes of secondary transmissions were noted in various HD units of Wan-Hua District (Taipei City), New Taipei City, Keelung, and many other areas in May 2021. In one of the HD units, one confirmed HD patient resulted in the propagation of disease into seven other HD patients and four medical staff members. The tremendous outcome of five deaths was noted in that episode. Effective prevention of the nosocomial spreading of SARS-CoV-2 among HD settings remains mandatory.

In spite of the exhibition of a high incidence rate of COVID-19 in HD patients (at Keelung) than the incidence of national citizens, this rate has remained low compared to international estimates of the HD population [10,28,29,30,31,32,33]. HD patients are more susceptible to SARS-CoV-2 infection because of older age, the coexistence of multiple comorbidities, and immune-suppressed status. Necessary and periodic visits to areas of high population density (public transportation or HD facilities) and close personal contacts (with caregivers, medical or nursing staff) make effective strategies to prevent COVID-19, such as social distancing or stay-home orders, difficult to execute for this vulnerable patient population [34,35,36]. However, our preparedness for fighting against the disease today is better than the experiences of the previous years in many other countries, where uncertainties arose at the beginning of the global pandemic. The learning of valuable information regarding disease prevention from various nephrology societies and experts over the past years has been extremely helpful. In addition, the relatively low confirmed domestic cases in our territory [9], early national level-3 alert measures, universal masking, early formulation of clinical practice guidelines for fighting of COVID-19 from the CDC, and TSN have all contributed to the lower incidence of our HD patients compared to the international burden of infection in the dialysis population [37]. From official guidelines, good indoor air ventilation, stringent environment cleaning, and disinfection are mandatory for HD settings. All HD patients must wear a mask during dialysis therapy. Other measures consist of stage-based, proactive screening using TOCC tracking, traffic control bundle and checkpoint hand washing, fixed caring model (including divided work team to care fixed group of patients) to minimize the staff-patient exposures [18]. The guidelines of TSN have further enforced tasks for leadership, education, preparedness, proactive management, and a recovery stage for fighting against COVID-19 [18]. We have carried forward and enhanced all of these recommendations in practice. For leadership, our rapid response team enabled immediate, confident, and transparent communication between staff; for education, periodic updates of informative posters and regular broadcasting to both the medical team and patients were performed in our HD unit every day; for preparedness and proactive management, a series of strategies focusing on space segmentation, access control, quarantine operations, and high-quality medical staff protection were built to optimize prevention control; ultimately, for the recovery stage, we stepped down our preventive plans, guided by using serial assessments of Rt values and regional prevalence rates, to restore the healthcare capacity and resource utilization into normal medical functioning. The COVID-19 vaccination program was very effective and has helped to achieve an inoculation rate of 82% to mid-July in our HD units. The application of all these policies was possible to contain COVID-19 transmission in our HD unit maintaining zero cases of infection to this day since the initiation of community outbreak in Taiwan.

The high mortality rate (66.6%) in COVID-19-infected HD patients in Keelung was peculiar. Analyses of the outcomes of COVID-19 infection have revealed overall mortality of 22.4% (ranged from 8.9 to 43.9%) in various HD patient cohorts reported in 2020 [10]. Common prognostic factors for mortality in COVID-19-infected HD patients included greater age, male gender, underlying comorbidities (cardiac or pulmonary disease, diabetes, and hypertension), and the use of mechanical ventilation, corticosteroids, or wheelchairs [36,38,39]. In addition to all these factors, the increased chest-thoracic ratio was also a poor prognostic predictor among our HD patients, highlighting the importance of fluid control and underlying cardiac performance in improving outcomes. Furthermore, the mortality of the second wave of the outbreak affected by COVID variants was higher than the initial global pandemic [40]. HD patients infected with SARS-CoV-2 variants B.1.362 (from Israel) were more likely to have critical COVID-19 (defined as a condition associated with organ failure requiring ventilation or hemodynamic support), shorter survival time, and poorer outcome, having a mortality rate as high as 57% than the non-variants COVID-19-infected HD patients [40]. Information regarding the outcome of HD patients infected with the SARS-CoV-2 variant B.1.1.7 (from the UK), the main virus strain in Taiwan, remains scarce. Large populational cohort studies revealed that patients infected with variants B.1.1.7 had a greater hospitalization rate, higher risk for intensive care, and higher 28-day mortality compared with patients with non-B.1.1.7 SARS-CoV-2 [41,42]. The emergency of SARS-CoV-2 variants has again emphasized the importance of infection control in HD settings because of the increased infectivity, virulence, and adverse outcome associated with these virus stains.

The HD unit of Chang Gung Memorial Hospital at Keelung is the largest renal care center in the region. We have successfully contained the COVID-19 infection and no secondary transmission was noted, despite the presence of one confirmed HD case infected from the household. National universal measures were rapidly implemented in addition to our specific preventive strategies from the beginning of the outbreak. Although we have provided a successful care model in preventing COVID-19 transmission in an HD facility, several works should be further addressed. First, many of our specific preventive plans were both resource- and time-consuming and may not be applicable to other private HD settings or areas of low prevalence rates. Our HD unit is a hospital-affiliated setting caring for approximately 500 HD patients, in which multidisciplinary infrastructure and medical resources are adequate to support all of the measures. The devotion of personnel to prevention controls may hamper a certain proportion of normal healthcare functions; however, through the use of high-quality PPE to protect our HD staff and the periodic evaluation of regional Rt values, it was capable of preserving medical manpower during the pandemic and allowed the gradual recovery of normal functioning leveraging extreme preventive policies. Second, the use of weekly rapid testing screening may have underestimated the exact incidence of disease due to the insufficient power of testing kits. The employment of double-checking of TOCC, clinical symptoms, and body temperature (at both entry of the hospital and at the door of the HD room) may help to identify suspected cases in a timely fashion. In addition, compliance and adherence to preventive plans represent key components of any disease control. Appropriate education, through postering and broadcasting, regular surveillance, and monitoring from the third-party administrative department may further consolidate the establishment of our preventive strategies. Finally, the emergence of new SARS-CoV2 variants and changes to immunity in the post-vaccination era may also arise new challenges in the care of HD patients in facing a global pandemic. COVID-19 remains less likely to be eliminated in the near future, persistent alerts, monitoring, and efforts in disease prevention may become new norms for the daily clinical care of renal patients.

## 5. Conclusions

In conclusion, we have demonstrated a real-world experience of combating the spread of COVID-19 in an HD unit situated in a hotspot area of the outbreak. This infection was associated with a high mortality rate. In addition to national universal prevention policies, specific preventive strategies such as space segmentation, access control, quarantine operations, and high-quality medical staff protection may enhance many tasks recommended by TSN to create a safe and clean medical area for HD patients and staff.

## Figures and Tables

**Table 1:** Clinical characteristics of maintenance hemodialysis patients in Chang Gung Memorial Hospital, Keelung Branch (*n* = 497).

	All HD Patients (*n* = 497)
Male, *n* (%)	263 (53)
Age, year	66.03 ± 13.28
White blood cell count (×1000/uL)	6.62 ± 2.87
Hemoglobin (g/dL)	10.06 ± 1.25
Platelet (×1000/uL)	190.47 ± 68.53
Albumin (g/dL)	4.00 ± 0.48
Aspartate transaminase (IU/L)	18.49 ± 10.31
Alanine aminotransferase (IU/L)	17.87 ± 14.74
Alkaline-P (IU/L)	92 (71–137) *
Total bilirubin (mg/dL)	0.38 ± 0.16
Cholesterol (mg/dL)	153.76 ± 36.27
Triglyceride (mg/dL)	151.77 ± 116.22
Creatinine (mg/dL)	9.40 ± 2.68
Uric acid (mg/dL)	6.43 ± 1.82
Na (meq/L)	138.10 ± 3.21
K (meq/L)	4.70 ± 0.83
Ca (mg/dL)	9.33 ± 0.90
P (mg/dL)	5.26 ± 1.55
Kt/V (Daugirdes)	1.67 ± 0.33
Normalized protein catabolic rate (g/kg/day)	1.08 ± 0.39
Ferritin (ng/mL)	496.46 ± 415.14
Transferrin saturation (%)	33.00 ± 14.51
Intact-parathyroid hormone (pg/mL)	285 (119, 672) *
Cardiac/thoracic ratio	0.52 ± 0.07
Ca × P product	49.17 ± 15.67

* Data are expressed as the median (interquartile range).

**Table 2:** Comparison between national and local incidence rates of SARS-CoV-2 infection and those of hemodialysis patients.

Districts	Confirmed Cases	Total Population ^#^	Incidence Rate (%)
National	14,804	23,816,775	0.062
Taipei City	4470	2,581,006	0.173
New Taipei City	6503	4,027,730	0.161
Keelung City	303	367,088	0.083
Overall HD patients at Keelung	9	1351	0.666
CGMH-HDR *	0 *	497	0.000

Abbreviations: CGMH-HDR, Chang Gung Memorial Hospital Keelung Branch hemodialysis room; HD, Hemodialysis. * Nosocomial infection. ^#^ Data source: National Center for High-performance Computing, https://covid-19.nchc.org.tw/city_confirmed.php (accessed on 1 August 2021).

**Table 3:** Specific COVID-19 prevention measures of the Dialysis Center of CGMH, Keelung Branch in 2021.

Strategies	Contents of Measures
Space segmentation	Use of solid compartment to separate the open room into cabins of 8–10 beds, using wood, strengthened glass, and acrylic as materials. Use of acrylic partitions in the nursing station to limit droplet dissemination. Fixed care bundle: fixed patients and caregivers in pre-established bed or seat location cared by a fixed nursing team using fixed clinical carts. Installation of monitoring and loudspeaker system in negative pressure isolation room to limit frequent entries and exits of personnel.
Access control	Spacing between exchanges of HD sessions to avoid overlapping of patients at entry and exit of HD room. Double TOCC checking at both entry of hospital (by reading of NHI card) and at the door of HD unit (by personal verbal and written confirmation). Double-checking of clinical symptom and body temperature at both entry of hospital and at the door of HD unit. Double hand alcohol disinfection at both entry of hospital and at door of HD unit. Remove tables and chairs in the waiting area and set up a position mark on the ground of entry with 1.5 m of distancing to avoid clustering. Control of the total number of people in the hospital and in a single elevator.
Quarantine operations	Postering and broadcasting (at entry of HD room, in the beginning of every HD session) of our HD preventive measures. Caregiver limitation (do not stay in treatment area, or fixed caregiver if assistance needed)
	Weekly rapid nasopharyngeal swab SARS-CoV-2 antigen testing for patients and fixed caregivers. Acceptance of new patients into our HD unit is prohibited during an outbreak. Triage algorithm should be followed for emergent cases (see Figure 3). Bedside vaccination program for all HD patients.
Staff protection	All medical and nursing staff wore high-quality PPE, including face shields, goggles, N95 masks, gloves, and waterproof isolation gowns. Staff entering the dialysis room must perform a QR code scan to confirm location and contact tracking.
	Provide the first dose of COVID-19 vaccine to all of the staff before the end of May. Staff who take care of confirmed patients should receive RT-PCR testing on a weekly basis.

All these measures were implemented in addition to those recommended by the Taiwan CDC and TSN. Abbreviations: HD, hemodialysis; TOCC, travel, occupation, contact, cluster history; NHI, national health insurance; PPE, personnel protection equipment; QR code, Quick Response code; COVID-19, coronavirus infection disease 2019; RT-PCR, real-time polymerase chain reaction.

**Table 4:** Characteristics of COVID-19 confirmed HD patients in Keelung City (*n* = 8) *.

	Total Patients (*n* = 8) *	Alive (*n* = 3) ^$^	Deceased (*n* = 5)	*p*-Value
Male, *n* (%)	3 (37.5)	1 (33.3)	2 (40)	0.714
Age, year	68.9 (60.88–97.00)	63.1 (60.88–64.30)	74.9 (61–97)	0.143
W.B.C. (×1000/uL)	4.20 (1.00–6.80)	5.30 (3.30–6.80)	4.20 (1.00–5.00)	0.250
Hb (g/dL)	9.55 (7.60–12.40)	8.20 (7.60–12.40)	10.5 (8.4–10.8)	0.571
Platelet (×1000/uL)	125 (39–225)	155 (76–173)	103 (39–225)	0.571
Albumin (gm/dL)	3.66 (3.06–4.15)	3.76 (3.27–3.91)	3.56 (3.06–4.15)	1.000
AST (IU/L)	25 (16–67)	26 (16–61)	24 (20–67)	1.000
ALT (IU/L)	17 (9–34)	31 (17–34)	12.5 (9–18)	0.114
Alkaline-P (IU/L)	110 (48–154)	128 (48–154)	110 (69–110)	0.700
Total Bilirubin (mg/dL)	0.35 (0.3–0.6)	0.3 (0.3–0.4)	0.4 (0.3–0.6)	0.393
Cardiac/thoracic ratio	0.59 (0.48–0.65)	0.55 (0.48–0.57)	0.61 (0.58–0.65)	0.036 *
Creatinine (mg/dL)	9.04 (4.00–15.18)	9.04 (6.39–11.74)	7.45 (4–15.18)	0.857
Na (meq/L)	137.5 (121–141)	138 (137–139)	135 (121–141)	0.393
K (meq/L)	3.70 (3.2–6.9)	4.60 (3.20–5.70)	3.70 (3.4–6.90)	1.000
Ca (mg/dL)	8.90 (8.50–11.10)	9.95 (8.8–11.10)	8.9 (8.5–10.0)	0.571
P (mg/dL)	5.00 (2.00–9.90)	4.40 (2.8–6.0)	5.0 (2.0–9.9)	1.000
Ferritin (ng/mL)	869.5 (41.9–6954.0)	877.0 (41.9–1100.0)	862.0 (82.3–6954.0)	0.786
Ca × P product	38.54 (20–88.11)	NA (31.08–52.80)	46.00 (20.00–88.11)	0.571
Ct value	17.50 (11.75–27.10)	17.53 (11.75–27.10)	17.47 (16.75–18.57)	1.000
CRP (mg/L)	36.62 (15.17–70.79)	17.2 (15.17–35.70)	52.81 (20.60–70.79)	0.071
LDH (U/L)	249 (185–355)	249 (185–355)	243.5 (219.0–290.0)	1.000
Lactate (mg/dL)	46.35 (7.80–116.60)	11.05 (7.80–14.30)	97.5 (78.40–116.60)	0.333
Procalcitonin (ng/mL)	0.535 (0.16–1.75)	0.51 (0.16–0.56)	0.56 (0.48–1.75)	0.393

Abbreviation: AST, aspartate transaminase; ALT, alanine aminotransferase; HD, hemodialysis; LDH, lactate dehydrogenase; WBC, white blood cell count; Ct value, threshold cycle for the measure of the concentration of target in the polymerase chain reaction for SARS-CoV-2 nucleic acid testing. * Overall confirmed 9 HD patients were noted in Keelung City. One patient died before admission to our hospital and only 8 patients were described in the present study. ^$^ One single patient in our HD unit transmitted from the household is one of these patients.

## Data Availability

Not applicable.

## Supplementary Materials

The following are available online at https://www.mdpi.com/article/10.3390/healthcare9091173/s1, Figure S1: Estimates of COVID-19 confirmed case number and 7-days moving average of confirmed cases. (A) National estimates, (B) in Taipei City and (C) in New Taipei City. Figure S2: Estimated and predicted time-varying reproduction number (Rt) of SARS-CoV-2 using national and subnational case counts reported in Taiwan Centers for Disease Control between 2 May 2021, and 30 June 2021. Estimation of time-varying Rt with 20%, 50%, 90% credible intervals using national data in Taiwan (A), Taipei City (B) and New Taipei City (C). Table S1: Clinical characteristics of non-COVID and COVID patients on maintenance hemodialysis in Keelung Chang Gung Memorial Hospital (*n* = 497).
