# Supplementary material for: Effective Preventive Strategies to Prevent Secondary Transmission of COVID-19 in Hemodialysis Unit: The First Month of Community Outbreak in Taiwan
Source: Healthcare (Basel). 2021 Sep 7;9(9):1173. doi: 10.3390/healthcare9091173 (PMC8467394; doi:10.3390/healthcare9091173)
Supplement: Supplementary file 1 [file healthcare-09-01173-s001.zip › healthcare-1364946-supplementary.pdf]

Table S1. Clinical characteristics of non-COVID and COVID patients on maintenance hemodialysis in Kee-lung Chang Gung Memorial Hospital (*n* = 497).

	**All HD patients (*n* = 497)**	**Non-COVID patients (*n* = 496)**	**COVID patient (*n* = 1)**
Male, *n*, (%)	263 (53)	263 (53)	0 (0)
Age, year	66.03 ± 13.28	66.04 ± 13.29	60.88
W.B.C. (x1000/uL)	6.62 ± 2.87	6.61 ± 2.87	6.80
Hb (g/dL)	10.06 ± 1.25	10.07 ± 1.25	8.20
Platelet (x1000/uL)	190.47 ± 68.53	190.51 ± 68.60	173
Albumin (g/dL)	4.00 ± 0.48	4.00 ± 0.49	3.76
AST (IU/L)	18.49 ± 10.31	18.49 ± 10.32	16
ALT (IU/L)	17.87 ± 14.74	17.87 ± 14.75	17
Alkaline-P (IU/L)	92 (71-137)*	92 (71-137)*	128
Total Bilirubin (mg/dL)	0.38 ± 0.16	0.38 ±0.16	0.3
Cholesterol (mg/dL)	153.76 ± 36.27	153.79 ± 36.31	139
Triglyceride(mg/dL)	151.77 ± 116.22	151.92 ± 116.30	82
Creatinine (mg/dL)	9.40 ± 2.68	9.40 ± 2.68	9.04
Uric acid (mg/dL)	6.43 ± 1.82	6.43 ± 1.82	6.9
Na (meq/L)	138.10 ± 3.21	138.10 ± 3.21	137
K (meq/L)	4.70 ± 0.83	4.70 ± 0.83	5.7
Ca (mg/dL)	9.33 ± 0.90	9.33 ± 0.89	8.80
P (mg/dL)	5.26 ± 1.55	5.26 ± 1.55	6.00
Kt/V (Daugirdes)	1.67 ± 0.33	1.67 ± 0.33	1.46
nPCR (g/kg/day)	1.08 ± 0.39	1.08 ± 0.39	1.03
Ferritin (ng/mL)	496.46 ± 415.14	497.43 ± 415.05	41.9
Transferrin saturation (%)	33.00 ± 14.51	33.03 ± 14.51	17.11
intact-PTH (pg/mL)	285 (119, 672)*	284.50(118.75, 672.75)*	440
Cardiac/thoracic ratio	0.52 ± 0.07	0.52 ± 0.06	0.48
Ca x P product	49.17 ± 15.67	49.17 ± 15.68	52.80

Abbreviation: WBC, white blood cell count; AST, aspartate transaminase; ALT, alanine aminotransferase; Hb, hemoglobin; i-PTH, intact-parathyroid hormone; nPCR, normalized protein catabolic rate. *Data are expressed as median (interquartile range)

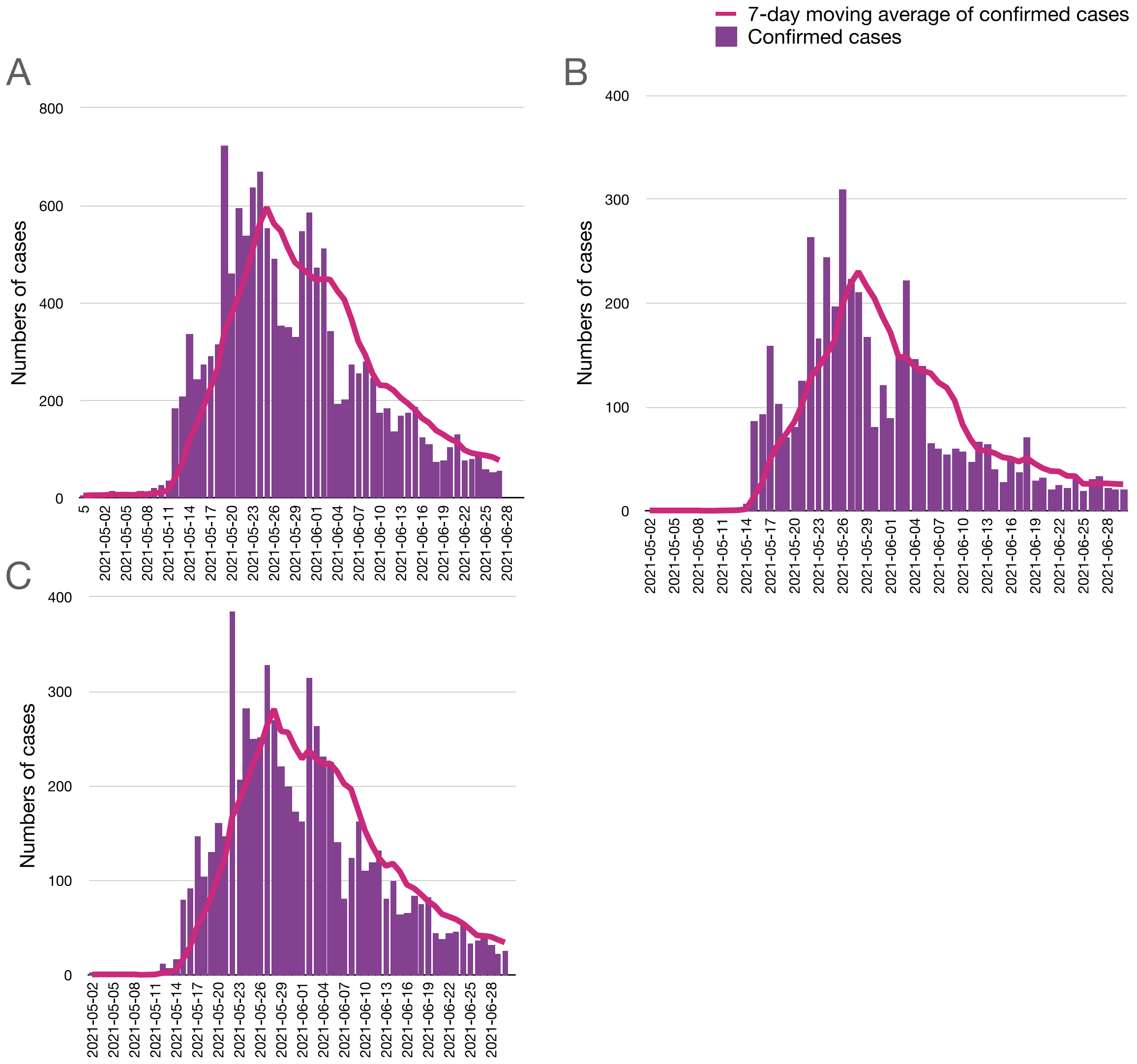

Figure S1: Estimates of COVID-19 confirmed case number and 7-days moving average of confirmed cases. (A) National estimates, (B) in Taipei City and (C) in New Taipei City.

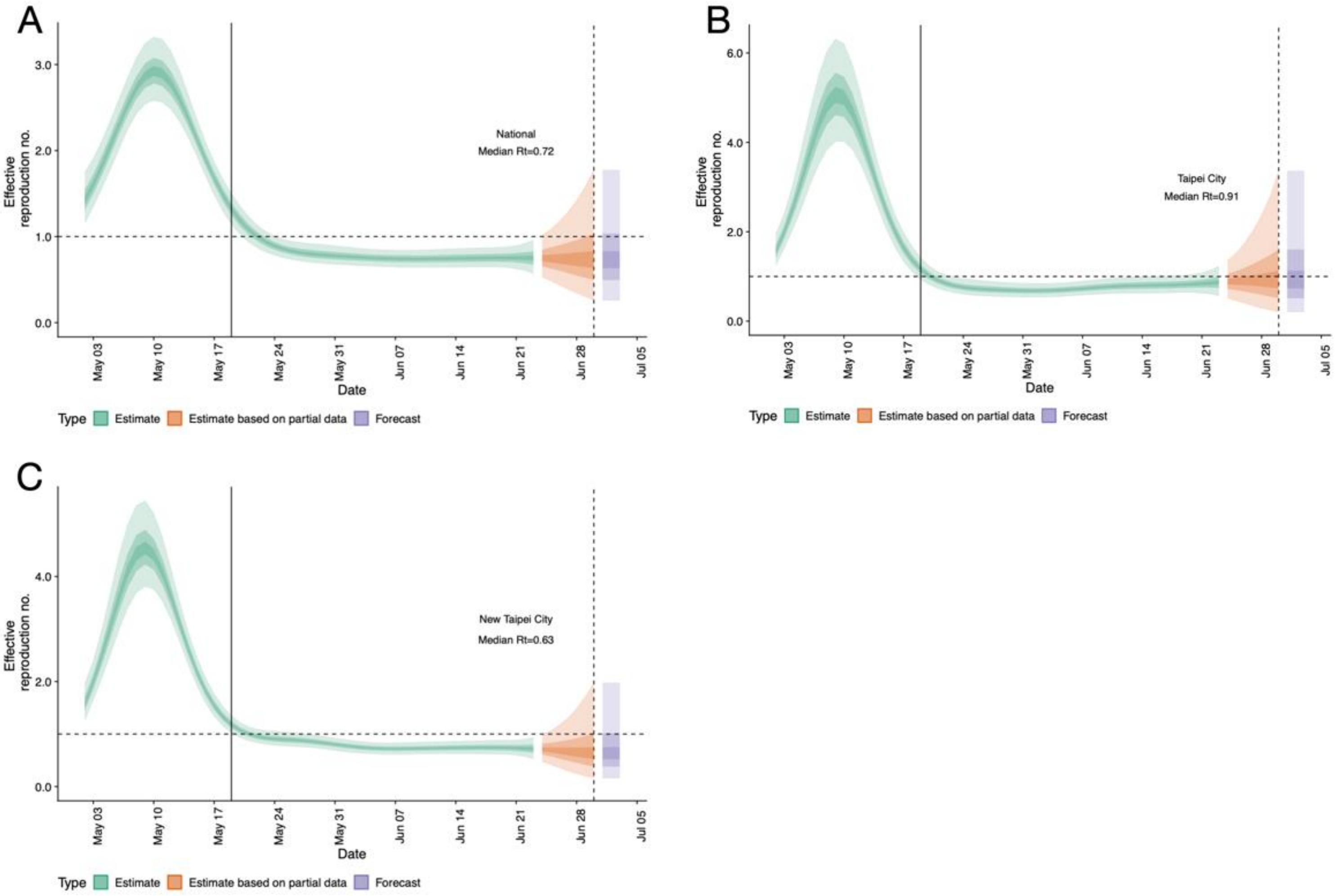

Figure S2: Estimated and predicted time-varying reproduction number (Rt) of SARS-CoV-2 using national and subnational case counts reported in Taiwan Centers for Disease Control between May 2, 2021 and June 30, 2021. Estimation of time-varying Rt with 20%, 50%, 90% credible intervals using national data in Taiwan (A), Taipei City (B) and New Taipei City (C).
